# Fibromyxoid Nephrogenic Adenoma Mimicking Renal Cell Carcinoma: A Diagnostic Pitfall in Fine‐Needle Aspiration Cytology

**DOI:** 10.1002/dc.70167

**Published:** 2026-06-28

**Authors:** Jodi Gedallovich, Adebola Adeniyi, Ankur R. Sangoi, Xiaohua Qian, Kelly Ernst

**Affiliations:** ^1^ Department of Pathology Stanford University School of Medicine Stanford California USA

**Keywords:** cytology, fibromyxoid, fine‐needle aspiration, immunohistochemistry, nephrogenic adenoma, renal cell carcinoma

## Abstract

Nephrogenic adenoma (NA) is a benign lesion of the genitourinary tract that may mimic malignancy both clinically and histologically. The fibromyxoid variant (FMNA), characterized by spindled cells in myxoid stroma, presents additional diagnostic challenges due to its rarity and overlapping features with renal cell carcinoma (RCC). A 72‐year‐old man with a history of nephrolithiasis and no prior urologic surgery was evaluated for a right renal mass. Fine‐needle aspiration yielded cloudy red fluid with scant epithelial cells and a fibromyxoid background. Immunohistochemistry showed positivity for PAX8, AMACR, CK7, and CA‐IX, leading to a pre‐operative diagnosis of RCC. Partial nephrectomy revealed a unilocular cyst with a solid component. Histology demonstrated fibromyxoid stroma with embedded spindled cells and focal tubular NA features; therefore, a final diagnosis of fibromyxoid nephrogenic adenoma was rendered. Fibromyxoid nephrogenic adenoma is a benign entity that can closely mimic RCC on cytologic and immunohistochemical grounds. Our findings underscore the diagnostic pitfalls in small‐volume cytologic samples. Awareness of the cytologic features of FMNA and cautious interpretation of immunostains are essential to avoid misdiagnosis and unnecessary treatment.

## 1 Introduction

Nephrogenic adenoma (NA) is a benign lesion of the genitourinary tract thought to arise from exfoliated renal tubular epithelial cells, often in the setting of prior injury [1]. Despite its non‐neoplastic nature, NA can pose significant diagnostic challenges by closely mimicking malignant neoplasms both clinically and histologically. The fibromyxoid variant, first described by Hansel et al. in 2007, is distinguished by focal elements of classic tubular nephrogenic adenoma and compressed spindled cells embedded in abundant fibromyxoid stroma [2]. In the largest case series of fibromyxoid nephrogenic adenomas (FMNA) to date, Li et al. identified 43 lesions predominantly in the bladder, prostate, and upper urinary tract of elderly men, most of whom had a history of prior urologic insult, nephrolithiasis, or instrumentation [3]. On cytologic examination, FMNA may present as bland glandular structures and spindled cells in a myxoid stromal background, features which can be easily misinterpreted as renal cell carcinoma, mucinous adenocarcinoma, or other malignant entities. The potential for misdiagnosis is compounded by the rarity of this variant and subsequent limited familiarity of pathologists with its features [1, 2, 3, 4, 5]. Additionally, the non‐specific immunohistochemical profile of FMNA including CK7, AMACR, CA‐IX, and PAX8 positivity can lead to confusion with other regional neoplasms. Here, we present a case of FMNA with special attention paid to the cytologic findings and the diagnostic pitfalls encountered.

## 2 Case Report

Our institution received a case in consultation involving a 72‐year‐old man with a past medical history significant for nephrolithiasis and diabetes mellitus who was evaluated by the urologic oncology service for an incidentally discovered right renal mass. He had no prior urologic surgical interventions. Computed tomography (CT) of the abdomen and pelvis revealed a circumscribed, solid and cystic, partially exophytic mass in the right kidney measuring 4.2 cm in greatest dimension. Notably, imaging studies performed approximately two years prior described the same lesion as a 5.7 cm renal cyst.

**FIGURE 1:**
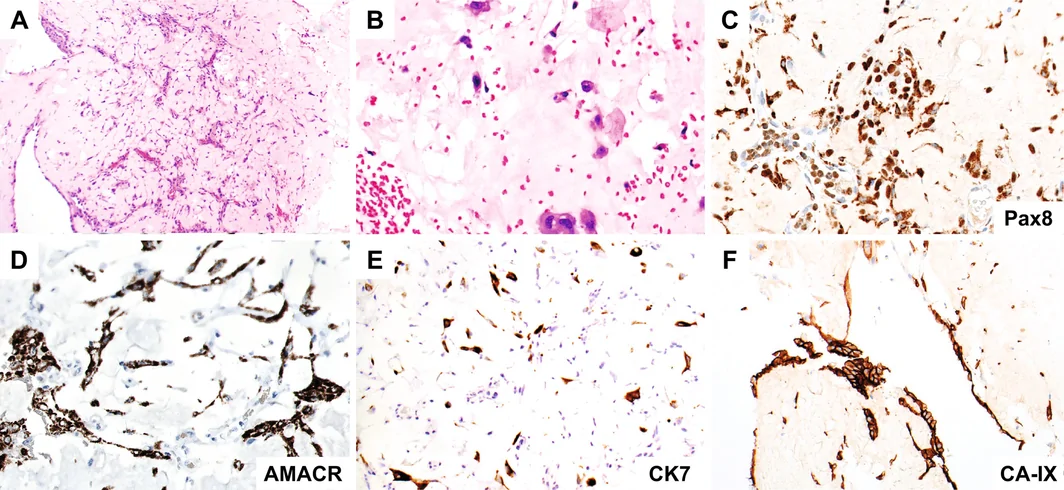
Histology and immunohistochemistry performed on fine needle aspiration material. (A and B) H&E stained sections, 4× and 20× magnification. (C) PAX8 immunohistochemistry. (D) AMACR immunohistochemistry. (E) CK7 immunohistochemistry. (F) CAIX immunohistochemistry.

Fine‐needle aspiration of the renal mass yielded 30 mL of cloudy red fluid. The specimen was submitted entirely for histologic evaluation. Cell block sections demonstrated sparse epithelial cells with irregular nuclear contours, variably prominent nucleoli, and minimal amounts of cytoplasm arranged in clusters and singly. Fibromyxoid change was present in the background. Immunohistochemical studies showed expression of PAX8 (diffuse, strong), alpha‐methylacyl‐CoA racemase (AMACR; mostly diffuse, moderate), and cytokeratin 7 (CK7) in lesional cells. Notably, lesional cells were positive for carbonic anhydrase IX (CA‐IX), including areas of complete membranous‐pattern reactivity. Cytokeratin 20, GATA3, CD117, and MelanA were negative (Figure 1). Based on these findings, a diagnosis of “consistent with renal cell carcinoma” was reported.

**FIGURE 2:**
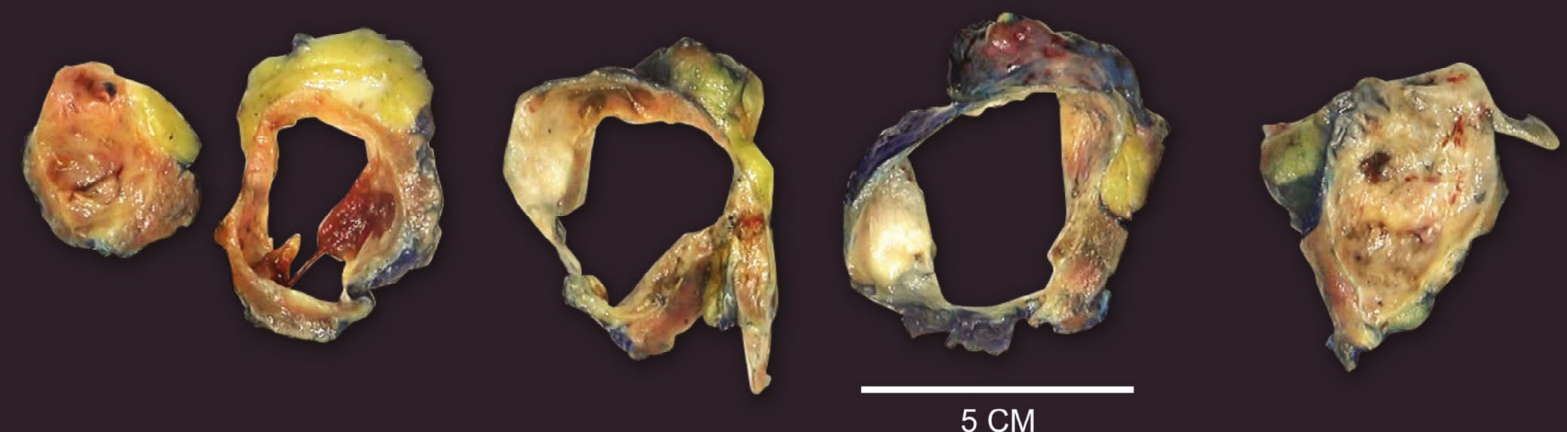
Gross images of fibromyxoid nephrogenic adenoma resection.

**FIGURE 3:**
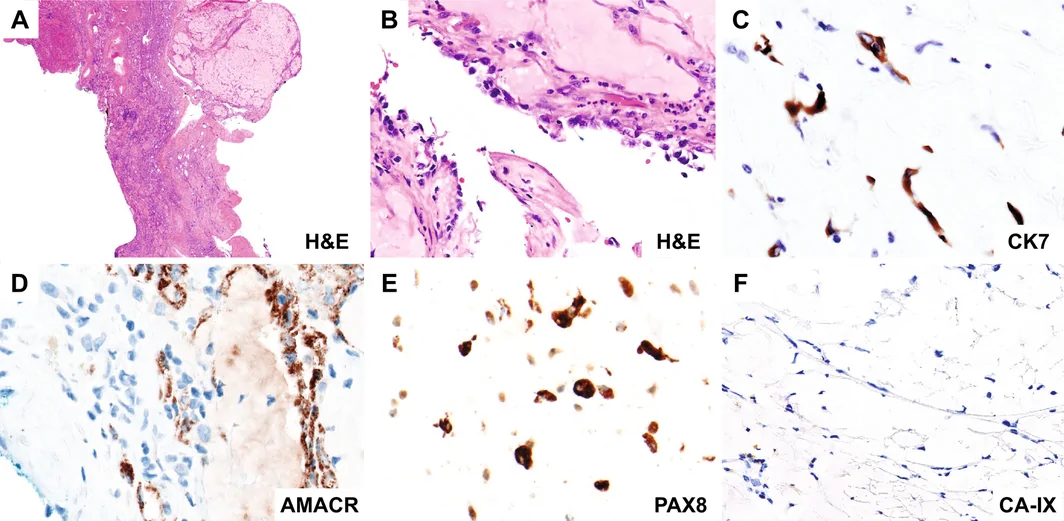
Histology and immunohistochemistry performed on surgical resection. (A and B) H&E stained sections, 4× and 20× magnification. (C) PAX8 immunohistochemistry. (D) AMACR immunohistochemistry. (E) CK7 immunohistochemistry. (F) CAIX immunohistochemistry.

The patient subsequently underwent robotic right partial nephrectomy. Gross examination revealed a unilocular cyst with a focally solid component measuring 0.8 × 0.8 × 0.3 cm located in the mid‐pole segment of the kidney (Figure 2). Histologic sections showed polypoid projections of fibromyxoid matrix containing embedded spindled cells extending into a cystic space. The spindled component showed diffuse positivity for PAX8 with patchy moderate expression of CK7 and AMACR with patchy but complete membranous pattern reactivity for CA‐IX (Figure 3) A final diagnosis of fibromyxoid NA was rendered. The patient showed no signs or symptoms of recurrence at follow‐up 6 months post‐operatively.

## 3 Discussion

In the genitourinary tract, fibromyxoid NA is typically discussed as a potential mimicker of prostatic adenocarcinoma and/or urothelial carcinoma, with limited data describing it as a diagnostic pitfall in the upper urothelial tract [3, 6]. FMNA may appear as a cystic structure or Bosniak category 3–4 lesion, raising clinical concern for renal cell carcinoma [5, 7]. Examination of fine‐needle aspirations of these lesions often produces sparsely cellular samples consisting of loose spindled cells in a background of prominent myxoid stroma, potentially leading to a false impression of low‐grade mesenchymal neoplasm, epithelial neoplasm, or even malignancy. In this case, the specimen was submitted entirely for histologic evaluation given the fluid volume of the sample. This case highlights important concepts in the present‐day practice of cytology: the utility and pitfalls of cell block analysis of fine needle aspiration specimens, and the relative unreliability of immunohistochemistry in small‐volume biopsies typical of cytology specimens. While no traditional cytologic preparations were made, cell block creation in the situation of a large sample volume with lesional cell paucity allows for maximum material preservation and immunohistochemical analysis. It is critical for cytopathologists, however, to treat cell blocks with prudence as the lesional architecture and cellular preservation are not reliably maintained. Given the substantial overlap of immunohistochemical features of FMNA with renal cell carcinomas, caution is advised regarding the interpretation of stains in limited cytologic preparations.

The immunohistochemical phenotype of FMNAs can have significant overlap with other renal neoplasms, most concerningly with renal cell carcinoma (RCC). To our knowledge, no reports in the literature discuss CA‐IX immunoreactivity in FMNA. We postulate that the positive CA‐IX staining seen in the initial fine‐needle aspiration sample, but not in the resection specimen, may be attributable to tissue hypoxia rather than neoplastic expression [8]. Positivity for PAX8 is expected in both entities. CK7 is generally positive in FMNA, as it is in papillary RCC, and as it can be in up to 15% of clear cell RCC [9, 10]. The literature contains reports of variable AMACR expression in NA. In one study, Gupta et al. reported positivity in 58% of 38 cases [11], however, work by Skinnider et al. suggests AMACR expression may be associated with tumor location; in the latter series, AMACR was expressed in 4/4 (100%) NAs involving the prostatic urethra but only in 3/16 (19%) NAs involving the bladder [12]. Mechanistically, AMACR positivity in NA may be related to the nature of the lesion itself; it is believed that they arise from exfoliated renal tubular cells, which typically show AMACR expression [12]. In the kidney, AMACR positivity can invoke a differential diagnosis of papillary renal cell carcinoma and clear cell renal cell carcinoma, with higher levels of expression associated with the former. Table 1 describes the profiles of key immunohistochemical markers for entities which should be considered in the differential diagnosis of fibromyxoid NA.

**TABLE 1:** Expected immunohistochemical profiles of differential diagnostic considerations.

	PAX8	CK7	CAIX	AMACR	CD117
Fibromyxoid nephrogenic adenoma	+	+	+	+	−
Clear cell renal cell carcinoma	+	−	+	+	−
Papillary renal cell carcinoma	+	±	+	+	−
Chromophobe renal cell carcinoma	+	+	−	−	+
Mucinous adenocarcinoma	+	+	−	−	−

Fibromyxoid NA is a rare benign entity of the urinary tract that has the potential to mimic various carcinomas when evaluated by cytology. Recognition of its fibromyxoid stromal background, mixed epithelial‐spindled appearance, and variable immunoprofile are critical for accurate identification. This case underscores the necessity of diagnostic caution and histologic correlation, when available, for atypical myxoid lesions that show expression of renal epithelial markers. Awareness of the fibromyxoid variant of NA and its cytologic features is critical for pathologists to avoid misdiagnosis and ensure the appropriate management of this benign process.

## Funding

The authors have nothing to report.

## Conflicts of Interest

The authors declare no conflicts of interest.

## Data Availability

Data sharing not applicable to this article as no datasets were generated or analysed during the current study.
